# Genito-Urinary Diseases

**Published:** 1904-10-22

**Authors:** 


(*To be continued.*)

### GENITO-URINARY DISEASES.

**Primary Carcinoma of the Prostate.**—George W. Hawley[1] has written on this subject. The disease often passes unrecognised *in vivo* and at necropsies. Post-mortem detection is extremely difficult without the microscope. Probably, therefore, the disease is more common than statistics would appear to show. All writers are unanimous that carcinoma of the prostate is much more common than sarcoma. The latter occurs usually before 30, and very occasionally after 50; it is therefore not so likely to be confused with senile hypertrophy. Cancer sometimes develops in prostates affected with senile enlargement. Albarran and Hallé found cancer in 14 out of 100 enlarged prostates. Similarly carcinoma may develop in an atrophied prostate. Prostatic cancer is rare before 50, but one case is recorded in which the patient was 29. In the majority of cases carcinoma of the prostate is small and of a slow-growing nature (Harrison). The growth usually begins in one or several small nodules, and these may remain stationary and yet give rise to extensive metastases. A portion or the whole of one or both lateral lobes, or more rarely the whole gland may be affected. Great hardness is characteristic, but soft areas have been noted in some cases, and breaking down and ulceration occur later. Local metastases appear in the form of small nodules beneath the mucosa of the urethra, trigone, seminal vesicles, or rectum, and these nodules rarely proceed beyond fixation of the mucous membrane. Metastases have also been found in the posterior portions of the corpora cavernosa. The favourite sites for secondary growths are the bones and lymphatic glands. Glands are not so frequently affected as in carcinoma of other organs. The three chief sources of osseous cancer are primary disease of the breast, of the thyroid, and of the prostate. No doubt the secondary growth in bones has often been overlooked. In a list of 22 cases of cancer of the prostate Kaufmann found skeletal involvement in 72 per cent. Usually many bones are affected. The vertebræ are most prone to suffer, then the ilium. Spontaneous fractures have occurred in one or two instances only. Internal organs are affected by metastasis fairly frequently. Persistent pain about the prostate in elderly men should awaken suspicion of the disease. Hæmaturia occurs in about 26 per cent. of cases. Hardness, tenderness, and a nodular contour of the rectal surface and margins of the prostate are the characteristics on rectal examination. When the bones are diseased myelocytes in considerable numbers may be found in the blood. Paraplegia may result from pressure on the spinal cord. Ovaison reports on 23 cases from the French clinics in which perineal prostatectomy was performed. In 10 of these cure remained permanent after more than four years; in three only was there recurrence; four died from the operation and six were lost sight of. Naturally, when metastasis has taken place to regional or remote organs, operation is contra-indicated, unless perhaps for the palliation of symptoms.

**Carcinoma of the Bulbous Urethra.**—J. Basil Hall[2] reports four previously unrecorded cases of this disease and gives a list of 21 cases, the only ones verified with the microscope which he has succeeded in finding after a careful search in the literature of the subject. Three cases are on record in which the growth arose primarily in Cowper's glands, and five cases recorded are of a doubtful kind because a microscopical examination was not made. According to Beck the disease occurs in men over 50 who have most commonly had previous disease of the urethra, *e.g.* gonorrhœal stricture. The most prominent feature of the cases is the formation of a hard, lobulated mass round the urethra. Micturition becomes difficult and painful. Hæmorrhage before and after micturition is common. The growth extends to the crura and corpora cavernosa and to the penile portion of the urethra. The glands in the groin get enlarged. Catheterisation becomes difficult and excites bleeding. In the four cases reported for the first time by Hall there was a marked absence of pain and hæmorrhage, and in two catheterisation was easy. The writer concludes that a differential diagnosis between simple induration and malignant disease is frequently impossible before operation, unless the perineal swelling is out of all proportion to that usually found associated with simple stricture. The possible existence of this disease should be borne in mind whenever a well marked perineal swelling exists. In half the cases the treatment has been palliative, in the other half radical. Of the former, all except one died within nine months. Of the ten cases treated radically the result is unrecorded in one, death occurred within nine months in four, another had recurrence in six months, and the remaining four were stated to be free from recurrence at twenty-one months, eleven months, ten months, and four months respectively. The growth spreads forwards and not to the prostate and tissues behind the triangular ligament, and the treatment by extirpation is worth trial.

**Sterility due to the Male.**—Egbert H. Grandin[3] has discussed this subject and emphasised the importance of gonorrhœa and syphilis as causes of sterility. Gonorrhœa is responsible not only for sterility, but also for many cases of pelvic disease in women and blindness in children. Man is responsible for race suicide; in 45 per cent. of the cases of sterility in women the man is the responsible party. In all cases of sterility the semen of the male should be examined, for in 6 to 8 per cent. of the cases the semen does not contain spermatozoa. Every young man should be instructed as to the risk he runs when he contracts gonorrhœa or syphilis. By proper educational methods the operating gynæcologist would be deprived of nearly 60 per cent. of his work and the human race immeasurably benefited.

[1] Annals of Surgery, June 1904, p. 376. [2] Ibid., March 1904, p. 375. [3] Abst. Med. Rec., March 26, 1904, p. 514.

